# Establishment and characterization of a new human pancreatic adenocarcinoma cell line with high metastatic potential to the lung

**DOI:** 10.1186/1471-2407-10-295

**Published:** 2010-06-16

**Authors:** Tatyana Kalinina, Cenap Güngör, Sabrina Thieltges, Maren Möller-Krull, Eva Maria Murga Penas, Daniel Wicklein, Thomas Streichert, Udo Schumacher, Viacheslav Kalinin, Ronald Simon, Benjamin Otto, Judith Dierlamm, Heidi Schwarzenbach, Katharina E Effenberger, Maximilian Bockhorn, Jakob R Izbicki, Emre F Yekebas

**Affiliations:** 1 Department of General, Visceral and Thoracic Surgery, University Hospital Hamburg, Eppendorf, Martinistrasse 52, 20246 Hamburg, Germany; 2 Department of Clinical Chemistry, University Hospital Hamburg, Eppendorf, Martinistrasse 52, 20246 Hamburg, Germany; 3 Hubertus Wald Tumorzentrum, University Cancer Center Hamburg, University Medical Center, University Hospital Hamburg, Eppendorf, Martinistrasse 52, 20246 Hamburg, Germany; 4 Institute of Anatomy and Experimental Morphology, University Hospital Hamburg-Eppendorf, Martinistrasse 52, 20246 Hamburg, Germany; 5 Institute of Pathology, University Hospital Hamburg, Eppendorf, Martinistrasse 52, 20246 Hamburg, Germany; 6 Institute of Tumor Biology, University Hospital Hamburg-Eppendorf, Martinistrasse 52, 20246 Hamburg, Germany

## Abstract

**Background:**

Pancreatic cancer is still associated with devastating prognosis. Real progress in treatment options has still not been achieved. Therefore new models are urgently needed to investigate this deadly disease. As a part of this process we have established and characterized a new human pancreatic cancer cell line.

**Methods:**

The newly established pancreatic cancer cell line PaCa 5061 was characterized for its morphology, growth rate, chromosomal analysis and mutational analysis of the K-*ras*, EGFR and p53 genes. Gene-amplification and RNA expression profiles were obtained using an Affymetrix microarray, and overexpression was validated by IHC analysis. Tumorigenicity and spontaneous metastasis formation of PaCa 5061 cells were analyzed in pfp^-/-^/rag2^-/- ^mice. Sensitivity towards chemotherapy was analysed by MTT assay.

**Results:**

PaCa 5061 cells grew as an adhering monolayer with a doubling time ranging from 30 to 48 hours. M-FISH analyses showed a hypertriploid complex karyotype with multiple numerical and unbalanced structural aberrations. Numerous genes were overexpressed, some of which have previously been implicated in pancreatic adenocarcinoma (GATA6, IGFBP3, IGFBP6), while others were detected for the first time (MEMO1, RIOK3). Specifically highly overexpressed genes (fold change > 10) were identified as EGFR, MUC4, CEACAM1, CEACAM5 and CEACAM6. Subcutaneous transplantation of PaCa 5061 into pfp^-/-^/rag2^-/- ^mice resulted in formation of primary tumors and spontaneous lung metastasis.

**Conclusion:**

The established PaCa 5061 cell line and its injection into pfp^-/-^/rag2^-/- ^mice can be used as a new model for studying various aspects of the biology of human pancreatic cancer and potential treatment approaches for the disease.

## Background

Pancreatic carcinoma is one of the most lethal neoplasms, with an overall 5-year survival rate < 5% [1,2]. Its incidence nearly equals mortality. This high mortality rate is due to an unusual aggressiveness, chemoresistance and early occurrence of metastatic disease. At the time of diagnosis, only a minority of about 20% of patients are in a non-metastatic stage of disease, which is the mandatory prerequisite for potentially curative surgery. To date, the molecular basis of this aggressive behaviour remains enigmatic [3,4], and further investigation of pancreatic cancer biology is of pivotal importance to provide new insights aiming at developing strategies for its prevention and treatment.

A major difficulty in studying pancreatic ductal adenocarcinoma (PDAC) biology is represented by the peculiar morphologic traits of the tumor. In fact, PDAC is characterized by a rich desmoplastic reaction. Tumor cells are often embedded into peritumoral, inflammatory alterations caused by tumor-associated, ductal obstruction [5,6]. For this reason, tumor cell lines solely represent a pragmatic tool consisting of clonal population of tumor cells with self-renewable features providing a basis for a variety of biologic and molecular experiments. Nevertheless, *in vitro *studies performed so far have distinct limitations. First, they are accomplished using a limited number of cancer cell lines, which have been cultured *in vitro *for a long time and may have altered their pheno- and genotypes. Second, many of the cancer cell lines used in these studies are derived from pancreatic cancer metastases and not from the original tumor. Third, several cell lines differ substantially from the clinical situation; they are often non-metastatic in conventional xenograft models. Although pancreatic cancer frequently metastasizes to regional lymph nodes and the liver in early tumor stages, only a few cell lines have been reported to spontaneously metastasize *in vivo *[7].

In the present study, we report biomolecular characteristics of a new human pancreatic adenocarcinoma cell line named PaCa 5061, which spontaneously metastasized into the lungs in a mouse xenograft model. We describe the cell line in terms of growth characteristics, phenotype, and genotype for their unique DNA and RNA profile using Affymetrix microarray technology as well as for specific alterations of relevant tumor-associated genes. Additionally, cytogenetic characteristics were accomplished by karyotype analysis.

## Methods

### Establishment of cell lines and culture conditions

Primary tumor tissues were taken from a 63-year-old male patient who underwent total pancreaticoduodenectomy for advanced pancreatic adenocarcinoma. Histopathological examination of the surgical specimen confirmed a low-differentiated adenocarcinoma of the pancreas, which was staged pT3, pN1 (7/43), G3, M0, R1. The patient died 6 weeks after surgery without having received any chemotherapy. Written informed consent of the patient for the removal of tissue samples for investigational purposes was obtained prior to surgery. The study was approved by the ethical committee of the Medical Council of Hamburg (Ärztekammer), Germany. Small fragments of tumor tissue with a diameter of 1 mm were obtained by mincing the tumor specimen with a scalpel. The fragments were enzymatically disaggregated after incubation with 0.5% collagenase type IV (Sigma-Aldrich, Steinheim, Germany) solution at 37°C on a rotary shaker. After 45 minutes, the solution was centrifuged at 700 g for 5 minutes, the pellet was collected, washed twice in cell culture medium (RPMI, Invitrogen, NY, USA) resuspended in complete medium (TUM), then plated into collagen-coated culture flasks (Becton Dickinson Labware, Bedford, MA, USA), and cultivated at 37°C in a humidified atmosphere with 5% CO_2_. The TUM medium was comprised of RPMI 1640 with Glutamax (Invitrogen, NY, USA) supplemented with 10% of fetal calf serum (FCS), 200 IU/ml of penicillin-streptomycin, 0.1 mg/ml gentamycin (Biochrom AG, Berlin, Germany), 50 nmol/ml of human transferrin (Sigma-Aldrich, Steinheim, Germany), 0.01 μg/ml of bovine insulin (Sigma-Aldrich, Steincheim, Germany), 0.01 μg/ml of recombinant human epidermal growth factor (Pepro Tech, London, UK), and 0.01 μg/ml of human basic fibroblast growth factor (Pepro Tech, London, UK). The growth medium was replaced every 4 to 7 days, and culture flasks were regularly checked for epithelial cells and fibroblast outgrowth. The cell line was cultured as monolayers in 25- or 75-cm^2 ^flasks, routinely passed by trypsinization, and maintained in complete culture medium. Cells at different culturing passages were stored in liquid nitrogen in culture medium containing 10% dimethyl sulfoxide. At this time the cells underwent 100 passages.

### Animals

The methods for carrying out the animal experiments were performed according to the UKCCR guidelines for the welfare of animals in experimental neoplasia [8]. Pathogen-free C57BL/6 pfp^-/-^/rag2^-/- ^mice (4 males and 4 females) aged 14-16 weeks were housed in filter-top cages: sterile water and food were given *ad libitum*. Mice weighed 25-30 g at the beginning of the experiment. All manipulations were conducted aseptically inside a laminar flow hood. One million PaCa 5061 cells (in 200 μl RPMI1640 medium without serum and antibiotics) were injected subcutaneously between the scapulae of each animal. The mice were killed when the tumor had reached approximately 10% of their total weight.

### Histology

After animals were sacrificed, primary tumors were excised and fixed in 4% buffered formaldehyde for 24 h, rinsed with phosphate buffer, dehydrated in a series of graded ethanol and embedded in paraffin. Sections of 5 μm thickness were cut and stained with haematoxylin and eosin (H.E.). To achieve a random distribution of each animal lung, the lungs were excised and fixed en block and cut into 1 mm thick lung slices. The slices were placed in warm agar and pressed down with a glass piston. After hardening of the agar these lung slices were processed paraffin-embedded as above. The agar blocks containing the lung slices were cut into 5 μm thick sections and the total number of sections of each lung was noted. In addition to every 10^th ^section, two series of serial sections (n = 30) out of the middle of the paraffin wax block were preserved for further immunohistological evaluation. Ten of the 10^th ^sections out of the middle of each paraffin wax block were H.E. stained. Metastases were counted in each of the ten stained sections under a microscope (Zeiss, Axioplan 2, 200×). The number of metastases for each mouse was calculated (mean number of metastasis × total number of sections - 20%), according to a formula established earlier [9]. To compare the *in vitro *grown cells with the *in vivo *grown tumors, cultured cells were pelleted and fixed with 4% formaldehyde and than embedded in agar (Agar Noble, Difco Laboratories, Detroit, MI, USA). Five μM sections were cut and mounted onto glass slides for IHC analysis.

### Immunostaining

Standard indirect immunoperoxidase procedures were performed with the peroxidase method according to manufacturer's protocol (HRP-AEC System, Cell and Tissue Staining Kit; R&D Systems, Minneapolis, MN, USA).

In order to confirm the human origin of even the smallest metastatic deposits, parallel sections to H.E. stained sections in which at least one metastasis was observed, were stained with a monoclonal pan-cytokeratin antibody, clone AE1/AE3 (Dako, Carpenteria, CA, USA). For IHC analysis the following antibodies were used: monoclonal CA 19-9, clone 116-NS-19-9, polyclonal rabbit CEA, and monoclonal EGFR, clone E30 (Dako, Carpenteria, CA, USA), monoclonal Mucin 4, clone 5B12 (Abnova, Taipei, Taiwan), monoclonal CEACAM-5, clone 487609 (R&D, Minneapolis, MN, USA) and monoclonal CEACAM6, clone 9A6 (Covance, Princeton, NJ, USA).

### Flow Cytometry

Cultured PaCa 5061 cells were trypsinized, washed and stained on ice with phycoerythrin (PE)- conjugated or fluoresceinisothiocyanate (FITC)-conjugated primary antibody without fixation. The following antibodies were used at the concentrations recommended by the manufacturer: CD44-FITC, clone B-F24 (Dianova, Hamburg, Germany); CEACAM1-PE, clone 283340 (R&D, Minneapolis, MN, USA); CEACAM5-FITC, clone C365D3(NCRC23) (AbD Serotec, Düsseldorf, Germany); CEACAM6-APC, clone 439424 (R&D, Minneapolis, MN, USA); EGFR-PE, clone EGFR.1 (BD, Heidelberg, Germany); EpCAM-PE, clone 1B7 (eBioscience, Frankfurt, Germany). The corresponding murine isotype controls were: IgG1-FITC (Miltenyi, Bergisch-Gladbach, Germany); IgG1-PE (eBioscience, Frankfurt, Germany); IgG2a-APC (R&D, Minneapolis, MN, USA); IgG2b-PE (Miltenyi, Bergisch-Gladbach). Flow cytometry was performed using a FACS CALIBUR flow cytometer (Becton Dickinson, Heidelberg, Germany). Expression profiles were analyzed using Win MDI 2.9 software.

### DNA fingerprinting analyses

The "Gene Print Fluorescent STR Multiplex Kit CSF1PO, TPOX, THO1 and vWA" (Promega, Madison, WI, USA) was used to compare patient's DNA to that of the cell line for excluding possible cross-contaminations with other cell lines. The PCR was carried out as recommended by the manufacturer and the PCR products were analyzed on an automatic sequencer (ABI PRISM 310 Genetic Analyzer) using the GenScan Analysis Software. The DNA fingerprinting profile of patient DNA was identical to this cell line. Comparative analyses of the fingerprinting profile of another cell line showed that this cell line was unique.

### Multicolor Fluorescence In Situ Hybridization (mFISH)

After 15 to 18 passages, the cultured primary cells were exposed to Colcemid (0,02 μg/ml) (Gibco-Invitrogen, Karlsruhe, Germany) overnight and subsequently harvested. Multicolor fluorescence in situ hybridization (mFISH) was performed using the 24XCyte color kit for human chromosomes (MetaSystems, Altlussheim, Germany) following the supplier's recommendations. Twenty-five well-conserved and complete metaphases were evaluated and karyotypes were described according to the International System for Human Cytogenetic Nomenclature (ISCN 2005).

### Affymetrix DNA SNP array analysis and data acquisition

DNA was extracted from punched tissue cylinders from frozen tumor samples or from pelleted culture cells according to the manufacturer's instructions of the QIAmp DNA Mini Kit (Qiagen, Hilden, Germany). DNA was further processed as described in the Affymetrix GeneChip Assay manual 6.0 (Affymetrix, Santa Clara, CA, USA). After hybridization to the GeneChip Genome-Wide Human single nucleotide polymorphism (SNP) Array 6.0, the microarray chips were washed and stained on an Affymetrix fluidics station. The chips were scanned using Affymetrix GeneChip scanner 3000 7G.

Raw data from scanned SNP arrays were acquired using the GeneChip Operating Software (Affymetrix). Quality of the data was checked as described in the GeneChip Mapping 6.0 Assay manual (Affymetrix). The data files were imported into the Chip software, and pre-processing and normalization were performed as described in the user manual. The resulting signal intensities were imported into R suite (R Development Core Team). Data were further processed with an especially created analysis tool using all measurements between 25^th ^and 75^th ^percentile as a reference for each DNA spot. Data were then modified to fit the input requirements of the DNAcopy package [10] of the Bioconductor suite [11]. The DNAcopy package was used to calculate and visualize segments with similar DNA content. Segments with higher DNA content than above calculated reference were classified as candidate regions for gene amplification.

### RNA isolation and GeneChip^® ^expression analysis

Total RNA from passage 27 of PaCa 5061 cells was isolated using the TRIzol^® ^reagent (Invitrogen, Groningen, The Netherlands) according to the manufacturer's protocol. The dried pellet was resuspended in 100 μl DEPC-water and used in the cleanup procedure with the RNeasy MinElute Cleanup Kit (Qiagen, Hilden, Germany). The cleanup process was followed by an overnight ethanol-precipitation, including 7.5 M NH_4_OAc and 2.5 volumes of absolute ethanol at -20°C. The RNA-pellet was dissolved in 10 μl RNase-free water. The RNA-concentration was measured on a NanoDrop^® ^ND-1000 Spectrophotometer (Peqlab, Erlangen, Germany) and the quality was checked with the Agilent 2100 Bioanalyzer (Agilent Technologies, Santa Clara, CA, USA).

Gene expression profiles of the whole human genome were generated by hybridizing either 5 μg of the isolated total RNA from the PaCa 5061 or 5 μg of human total pancreas RNA (Stratagene, La Jolla, CA, USA) serving as healthy control RNA on GeneChip^® ^Human Genome U133 Plus 2.0 Arrays (Affymetrix, Santa Clara, CA). The analysis was performed according to the One-cycle cDNA synthesis protocol for GeneChip^® ^Expression Analysis Manual (Affymetrix), and the achieved data were analyzed using an Affymetrix GeneChipOperatingSoftware (GCOS) 1.4 and scaled to a default target signal value of 150. Absolute and comparative analysis was performed using the Affymetrix MAS 5.0 algorithm. Annotations were further analyzed with interactive query analysis at http://www.affymetrix.com.

### Cell viability analysis

To determine the drug sensitivity of the PaCa 5061 cells, they were incubated with different concentrations (1-10 μmol) of Gemcitabine (Lilly Deutschland GmbH, Giessen, Germany), Cetuximab (Merck, Darmstadt, Germany), Fluorouracil (5-FU) (Sigma-Aldrich, Steinheim, Germany), or Gefitinib (Astra Zeneca, Macclesfield, Cheshire, UK), respectively. Cell proliferation was determined by the CellTiter 96^® ^AQ_ueous _One Solution Cell Proliferation Assay (Promega, Madison, WI, USA). Briefly, the cells were seeded into 96-well culture plates (5000 cells/well) in TUM medium and allowed to attach at 37°C and 5% CO_2 _in humidified air for 24 h. The medium was replaced by the drug containing medium for a subsequent 72 hrs. Each experiment was performed in quadruplicate. At the end of the incubation time, 20 μl of substrate was added to every well. The reaction mixture was incubated at 37°C for 2 h, and the absorbance was measured at 490 nm. Values for control cells were considered as 100% viability. Every measurement was performed at least in three independent experiments.

### Data analysis

Experiments presented in the figures are representative of three or more different repetitions. The data are presented as the mean values ± SE.

## Results

### Establishment of cell lines

**Figure 1:**
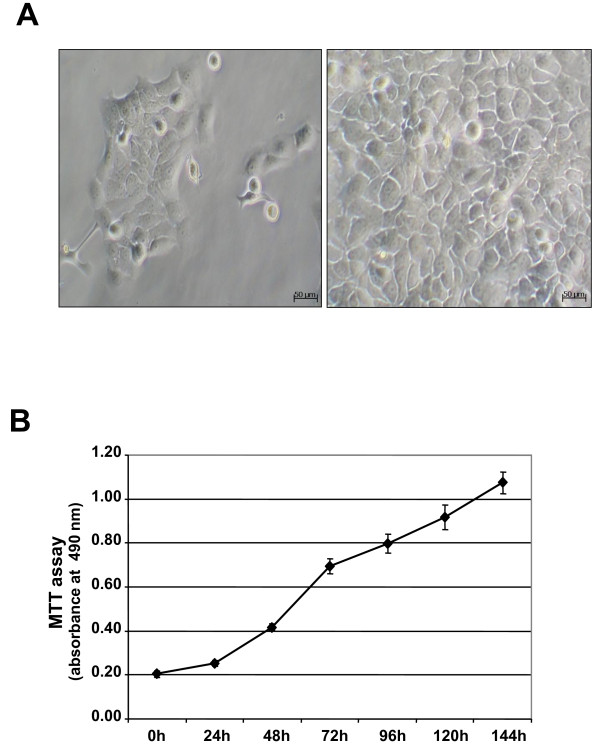
**Cell morphology and growth characteristics of PaCa 5061 cells**. **1A**. Morphological examination of non-confluent and confluent PaCa 5061 cells by phase contrast microscopy. The cells grew in a monolayer with polygonal shape, epithelial morphology and large nuclei. **1B**. Growth curve of PaCa 5061 in culture. Proliferation of cells (1000/well) was determined every 24 hours and relative growth rates were analyzed over time (0-144 h) by MTT assay. The results shown are for an experiment representative of three independent assays.

**Figure 2:**
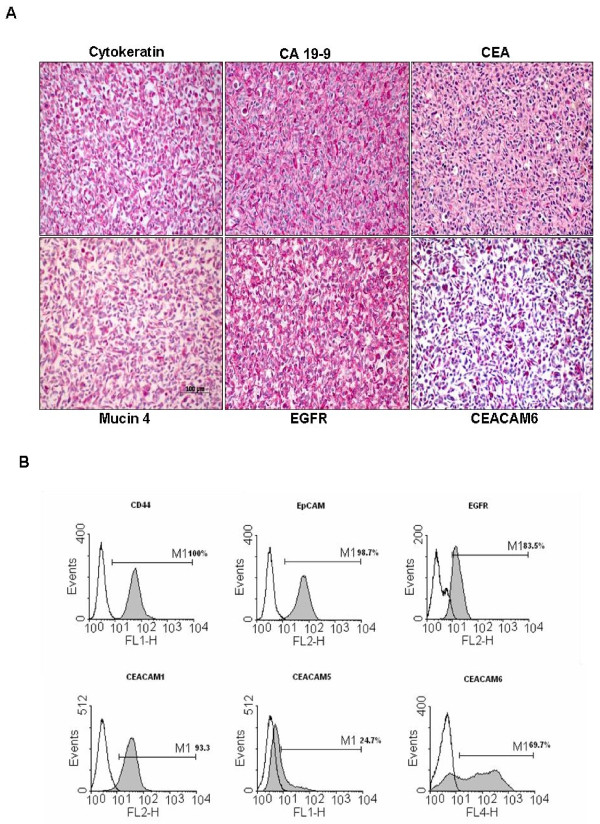
**Immunostaining and cell surface expression of epithelial/pancreatic markers and overexpressed proteins in PaCa 5061 cells**. **2A**. The formaldehyde fixed and agar embedded cells were immunostained for the presence of pancreatic cancer markers CA 19-9 and CEA respectively, as well as for cytokeratin as epithelial cell marker proteins (upper panel). Several on RNA-level overexpressed genes (Microarray) were chosen. To confirm protein overexpression of selected amplified genes in PaCa 5061 cells immunostaining was performed for Mucin4, EGFR and CEACAM 6 (lower panel). **2B**. FACS profiles of PaCa 5061 cells. Cell surface expression of CD44, EpCAM, EGFR, CEACAM1, CEACAM5 and CEACAM6 were obtained with specific antibodies as in materials and methods. Each histogram shows cell surface expression of the corresponding marker (filled curves) and the irrelevant, isotype-matched antibody (open curves).

A new pancreatic carcinoma cell line designated PaCa 5061 was generated from a resected PDAC. Within the first 2 weeks, the tumor cell clusters adhered to the surface of the cell culture flasks and gradually formed cell colonies. Initially, contaminating fibroblastic cells proliferated and surrounded the tumor cell colonies. During serial passages, the number of fibroblastic cells gradually decreased and was replaced entirely by tumor cells (Figure 1A). All cells adhered tightly to the bottom of the cell culture flasks in a monolayer, and were characterized as polygonal epithelial-like cells with large nuclei containing several nucleoli. The appearance of PaCa 5061 cells was polygonal. The population doubling time ranged from 30 to 48 hours (Figure 1B). The epithelial phenotype was confirmed by the positive immunoreactivity for cytokeratin and the expression of the pancreatic tumor markers CEA and CA 19-9 (Figure 2A). The same pattern of immunoreactivity was observed in the primary tumor of patient. FACS analysis of PaCa 5061 cell population revealed homogeneous expression levels for EpCAM (98.7%), CD44 (100%), EGFR (83.5%) as well as CEACAM 1 (93.3%) whereas heterogeneous expression levels was observed for CEACAM 5 (24.7%) and CEACAM 6 (69.7%) (Figure 2B).

### Multicolor fluorescence in situ hybridization and molecular genetic analyses

**Figure 3:**
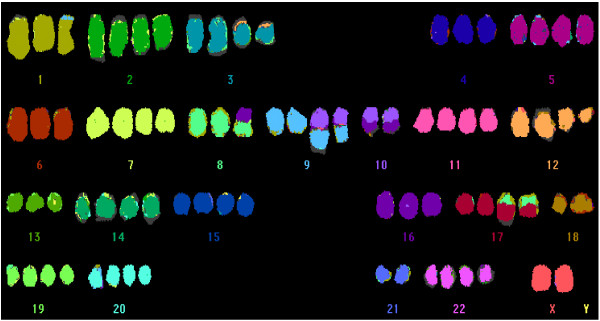
**mFish analysis of PaCa 5061 cells**. **mFish analysis of PaCa 5061 cells**. Representative karyotype of PaCa 5061 cells. Multiple numerical and unbalanced structural aberrations were observed in the majority of cells. *Note*. Whole-chromosome losses of 18, 21 and Y, as well as, gains of chromosomes 11 and 20 are frequently found in pancreatic cancer.

An abnormal hypertriploid complex karyotype was detected in all analyzed metaphases of the PaCa 5061 cell line (Figure 3). The karyotype was as follows: 78 < 3n+ > ,XX,-Y,der(1)t(1;9)(p35;?),+2,del(2)(p21)x2,+3,der(3)t(3;12)(p11;?)x2,+5,+7,del(7)(q22)x2,der(8)t(8;16)(p11;?),+del(9)(p13),del(9)(p13),der(9;10)(q10;q10)x2,10,der(10)t(10;16)(q11;?)x2,+11,+12,del(12)(q15)x2,+14,+15,+17,der(17)t(8;17)(?;p11)x2,-18,+19,+20,-21,+22[cp25]. Almost all chromosomes were affected by numerical or structural changes. Numerical abnormalities were found more frequently than structural rearrangements, and chromosomal gains were more frequent than losses. Six unbalanced translocations involving chromosomes 1, 3, 8, 9, 10, and 17 were seen, however, balanced translocations were absent. The identifiable breakpoints of the unbalanced translocations were located in the chromosomal regions 1p35, 3p11, 8p11, 9q10, 10q10/q11, and 17p11. Whole chromosome gains of chromosomes 5, 11, 14, 15, 20, and 22 as well as partial chromosome gains of 2, 3, 7, 8, 9, 12, 16, and 17 were observed in this cell line. Whole chromosome losses affected chromosomes Y, 10, 18, and 21, and partial losses were detected for chromosomes 2, 3, 7, 8, 9, 12, and 17.

The cytogenetic findings were correlated and extended by the DNA-SNP array data. As expected, imbalances targeting small chromosomal regions remained beyond the limits of detection of mFISH.

An activating mutation was found in codon 12 of the K-*ras *gene. The PaCa 5061 had a G to A homozygous transition (GGT(gly) > GAT(asp)), while no inactivating mutations were found in TP53, exon 4, 5, 6, 7, 8, 9 and EGFR exon 18-21. A polymorphism at codon 72 (CGC-arg) of p53 was also noticed in PaCa 5061 cells.

### Copy number variations and differential gene expression

**Figure 4:**
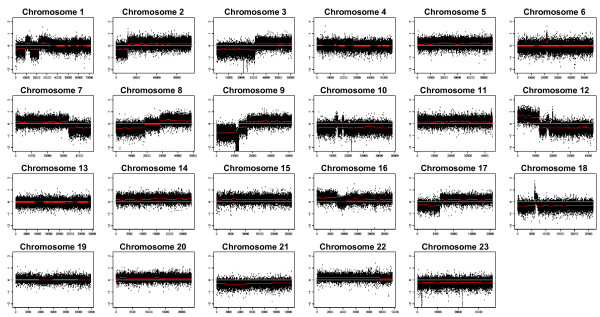
**Whole-genome DNA profile of PaCa 5061**. The DNA profile performed by Affymetrix GeneChip hybridization shows a considerable number of large chromosomal alterations. Detailed candidate regions of gene amplification and affected genes are listed in Table 1.

The Affymetrix 6.0 SNP array was used to identify the genes amplified in PaCa 5061 cells and primary tumor DNA. The DNA profile of PaCa 5061 cells and the primary tumor of the same patient are shown in Figure 4. Although there are much stronger DNA copy number variations (CNV) in the cell line as compared to the primary tumor sample, the general patterns of CNV were highly similar. The DNA profile showed a considerable number of large chromosomal alterations, including deletions involving at least parts of chromosomes 1p, 2p, 3p, 7q, 8p, 9p, 10, 12q, 17p, 18, and 21. In addition, gains were found at chromosomes 8q, 12p, 14, 16, 17q, 4 and 22. Chromosomal areas of high level gene amplification were found at 2p22, cen6, 7q21, 10p12, 10q11, 12q13, 12q14, and 18q11. The exact amplicon positions and affected genes are summarized in Table 1.

**Table 1:** Amplicon positions and affected genes

Localization	Amplicon position (pb)	Copy number variations	Putative target gene (s)
2p22	31168964-32709448	11	MEMO1

7q21	88495972-89700934	2	STEAP2

10p12	26552050-30207158	17	RAB18

10q11	41956473-44148144	11	RET

12q13	50980560-56120694	143	ERBB3 IGFBP6 STAT2 STAT6 MMP19 MAP3K12

12q14	62947641-64014306	7	TBK1

18q11	17336609-18060812	6	GATA6 MIB1 RIOK3

The gene expression profile of the PaCa 5061 cell line was compared to the RNA expression pattern obtained from normal pancreas. In total, 7012 genes were overrepresented more than 1.5-fold and 4212 were down-regulated at least 1.5-fold. In general, the down-regulated genes represent the pancreas-specific genes involved into metabolic pathways such as glycolysis and gluconeogenesis. The suppression of these genes could be associated with the dedifferentiation of pancreatic cells during malignant transformation. Most of the upregulated genes represent different proteins associated with cell proliferation and resistance to apoptosis, as growth factor receptors (EGFR, IGF-IR), different kinases (AKT, MAPK), and growth factors (amphiregulin, HBEGF, epiregulin, VEGF). Some pro-angiogenic factors (IL18, IL15, MMP10, MMP28) as well as cell adhesion molecules (CEACAM 1, CEACAM 5, CEACAM 6) were also overexpressed. The fold changes of some selected genes are shown in Additional file 1. The highest overexpression concerned the surface protein Mucin 4 (MUC4), which is involved in the regulation of cell adhesion. As expected, some amplified genes (MEMO1, IGFP6, TBK1, MIB1, RIOK3), determined by the DNA chip array demonstrated high level of RNA overexpression as well.

### Tumorigenicity and metastatic ability of the PaCa 5061 cells

**Figure 5:**
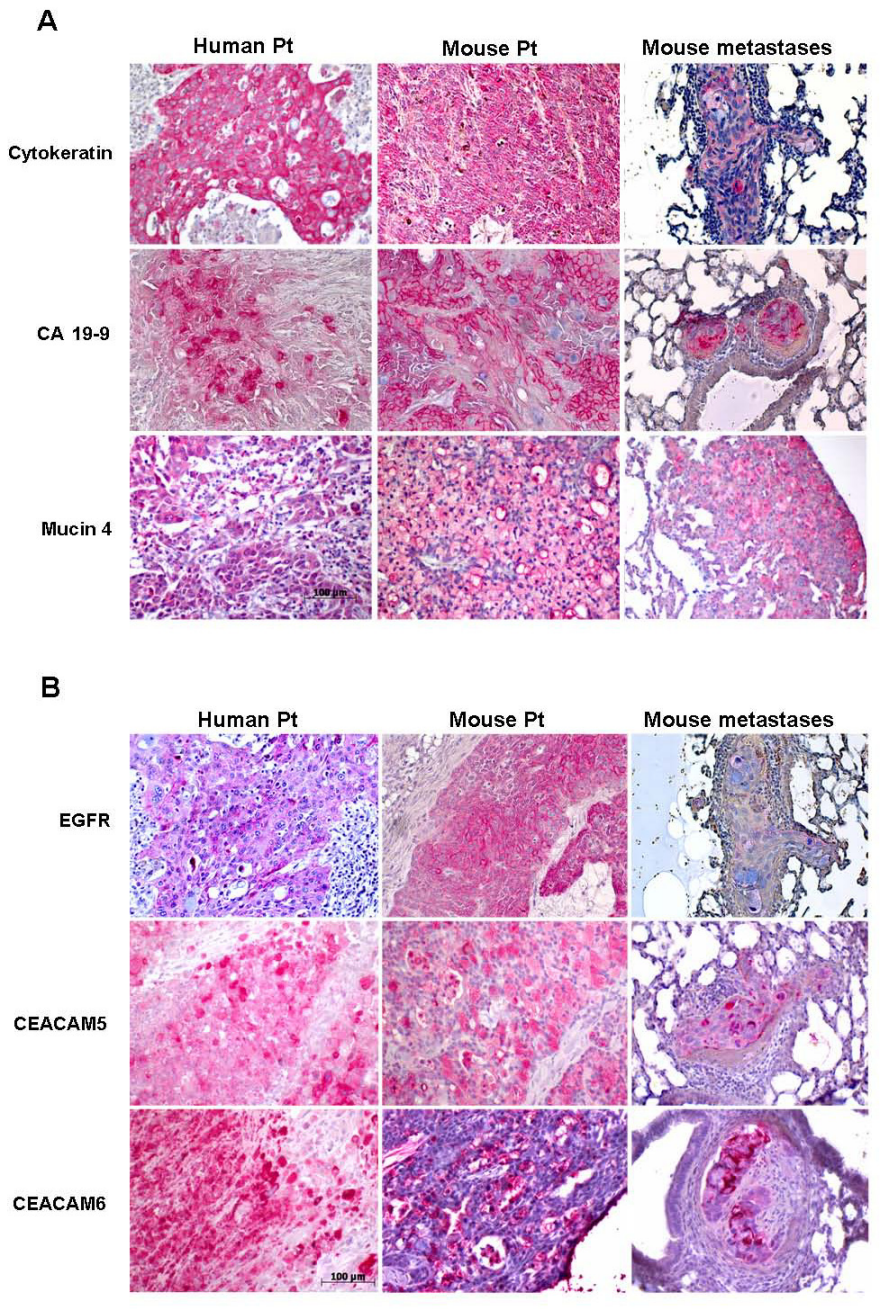
**Immunostaining of primary Patient Tumor in comparison to xenografts of PaCa 5061 cells in pfp^-/-^/rag2^-/- ^mice**. Primary patient tumor as wells as primary mouse tumor and corresponding lung metastasis were immunostained. **5A**. Human primary tumor, mouse tumor and corresponding lung metastasis are positive for cytokeratin and pancreatic cancer cell markers CEA and CA 19-9 respectively. Positive immunostainings for overexpressed Mucin4 are shown, confirming the Microarray data. **5B**. Patient primary tumor as well as mouse tumors are highly positive for EGF receptor as well as for CEACAM 5, and -6 adhesion molecules, confirming the Microarray data. Remarkably, the pattern of the staining intensity as well as the expression pattern showed a high level of similarity between human and mouse primary tumor.

To assess the *in vivo *tumorigenic potential of PaCa 5061 cells, 1×10^6 ^cells were transplanted subcutaneously between the scapulae of immunodeficient pfp^-/-^/rag2^-/- ^mice. After 14-23 weeks, all mice developed local tumors at the site of injection. No macroscopically detectable metastases were detected at necropsy, however, histological examination of animals´ lungs revealed the presence of numerous metastases in 6 out of 8 mice (Table 2). The median number of metastases was 724 with a range from 24 to 1948. The pancreatic carcinoma cell markers (cytokeratins, CA 19-9) as well as the other overexpressed proteins identified in PaCa 5061 cell line (MUC 4, EGFR, CEACAM 1, -5, and -6) were analyzed by IHC in mouse primary tumor and corresponding metastasis and compared with the expression of this marker in the primary tumor of the patient. The pattern of the staining intensities as well as the expression patterns showed a high level of similarity between human and mouse primary tumor (Figure 5A and 5B).

**Table 2:** Number of primary tumors/metastasis in mouse lung resulted from transplanted PaCa 5061 cells in vivo

Tumor Incidence	Tumor weight (g) Median (range)	Time of tumor growth (days) Median (range)
**8/8**	**2.35 (1.38 - 4.4)**	**101 (99 - 163)**

**Metastasis Incidence**	**Median **(range)	
	
**6/8**	**724 (24 - 1948)**	

### Drug sensitivity

**Figure 6:**
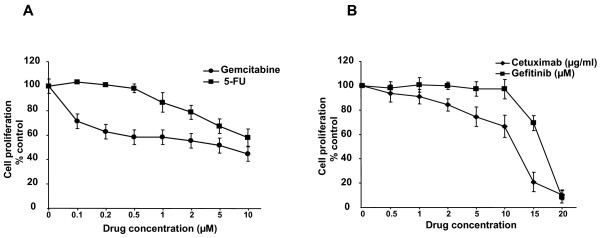
**PaCa 5061 cell viability following Gemcitabine, 5-FU, Cetuximab, and Gefitinib treatment**. Cell proliferation following drug treatment for 72 h was estimated by MTT test. **6A. **Dose-dependent inhibition of cell proliferation in Gemcitabine and 5-FU treated cells as well as in Cetuximab and Gefitinib treated cells (**6B**). Quantitative values are means ± SEM from 3 independent experiments performed in quadruplicate.

We examined the cellular sensitivity of PaCa 5061 cells to different drugs *in vitro *using the proliferation assay. PaCa 5061 cells were treated with increasing concentrations of the chemotherapeutic drugs Gemcitabine (0.1-10 μM) and 5-FU (0.1-10 μM). PaCA 5061 cells exhibited native resistance to both drugs and inhibiton of cell proliferation to 50% (IC50) was achieved with 10 μM for Gemcitabine and > 10 μM for 5-FU respectively (Figure 6A). As PaCa 5061 cells were characterized by an elevated expression level of EGFR, we blocked EGFR activation by a tyrosin-kinase inhibitor (Gefitinib) or EGF binding by a monoclonal EGFR antibody (Cetuximab). PaCa 5061 cells showed robust resistance to these drugs, and IC50 was achieved with > 15 μM for Gefitinib and > 10 μg/ml for Cetuximab (Figure 6B).

## Discussion

The biology of PDAC is poorly understood. Primary tumor cell lines serve as an available preclinical research tool for a better understanding of several tumor-related pathological aspects and the evaluation of anticancer agents [12]. However, the impact of such studies is often limited because information concerning the original tumor from which the cell lines were derived is scarce. Many mammalian cell lines, serving as the basic platform for investigations in biomedical research, have been cultured for more than 40 years. The proportion of research papers flawed by the use of misidentified and cross-contaminated cell cultures accounts for approximately 15-25% [13,14]. In this study we complied with the evident need for new cancer cell lines which reliably reflect the clinical features of pancreatic cancer such as molecular biomarkers and metastatic behaviour.

The first report on successful cultivation of a human pancreatic cancer cell line dates back to 1963 [15]. Since then, well over 60 human pancreatic cancer cell lines have been reported [16]. Only a few of these cell lines (Capan-1, SUIT-2, SUIT-4 and PCT-1) have been described to metastasize spontaneously *in vivo *into the regional lymph nodes or distant organs such as liver, lung or peritoneal cavity in nude mouse subcutaneous xenograft models [17]. In this context Loukopoulos *et al*. have previously shown that orthotopic injection of such cell lines in SCID mice resulted in extensive local tumor growth and metastatic spread [18].

It is widely accepted that xenograft mouse models which feature cancer cells growing in their natural location resemble the clinical situation closer than subcutaneous mouse models including (i) extensive local tumor growth, (ii) metastases to the liver and regional lymph nodes, and (iii) distant metastases to the diaphragm and mediastinal lymph nodes. Although orthotopic implantation models are preferred over subcutaneous models, previous studies have shown that orthotopic implantation has more potential for complications and is often affiliated with a varying rate of tumorigenicity and metastasis formation *in vivo *[19]. Nevertheless, subcutaneously injected cancer cells grow well at heterotopic sites but they infrequently adopt the real clinical situation as they are often non-metastatic. Therefore, our newly established PaCa 5061 cell line is one of very few cell lines capable to spontaneously metastasize into the lung of pfp^-/-^/rag2^-/- ^mice with high efficiency after subcutaneous transplantation. Hence, this cell line reliably reflects the clinical situation and is therefore capable to investigate the complex biology of pancreatic cancer progression, cancer cell dissemination and metastasis formation as well as chemoresistance of this deadly neoplasia *in vivo*, even without application of an orthotopic implantation model.

The PaCa 5061 cell line, derived from a PDAC, was immunoreactive for general epithelial cell markers such as cytokeratins, the epithelial cell adhesion molecule (EpCAM), MUC4 which is involved in the regulation of cell adhesion and membrane-bound glycoprotein CD44. Furthermore, more specific pancreatic tumor markers, such as CA 19-9 and CEA were displayed by the cells confirming their organic origin. The ability of these cells to form tumors in pfp^-/-^/rag2^-/- ^mice proved their tumorigenic potential. In addition, the highly invasive and aggressive behaviour of these cells was demonstrated by their ability to form metastases in 75% of the test animals.

The histopathological profile of the primary xenograft tumor obtained after injection of PaCa 5061 cells strongly resembles that of the original tumor. This finding was in line with previous results reported from ovarian carcinoma, where patient samples and mouse xenotransplants showed high concordance [20].

Traditional chromosome analyses provided important information on chromosomal aberrations in pancreatic cancer and revealed a high degree of ploidy with many chromosomal abnormalities [21]. Cytogenetic characterization of our new cell line performed by mFISH analysis showed a hypertriploid complex karyotype with multiple numerical and unbalanced structural aberrations. According to previous studies, the recurrent chromosomal abnormalities seen in pancreatic cancer, such as whole-chromosome losses of chromosomes 18, 21 and Y as well as gains of chromosomes 11 and 20 were also present in this cell line [22]. Furthermore, we observed partial gains of chromosomes 8q and 12p, and partial losses of 9p, 12q, and 17p, similar to previously reported PDACs [23-25].

An increasing number of studies have demonstrated that multiple genetic alteration steps are associated with malignant progression in PDAC. Among these, the activation of the K-*ras *oncogene and inactivation of the p53 tumor suppressor gene have been proposed to play a pivotal role in PDAC progression [26,27]. Mutation of K-*ras *gene was also detected in PaCa 5061 cells, in the xenografted tumors as well as in primary human tumor samples (activating mutation at codon 12). This finding is consistent with previous studies which have reported that the codon 12 mutation from GGT(Gly) to GAT(Asp) in the K-*ras *gene is one of the most frequent mutations in pancreatic cancer [28]. In contrast, neither inactivating mutations of the p53 gene nor mutations that concerned exons 18-21 of the EGFR gene were found.

A number of studies on pancreatic cancer have used DNA microarrays in order to determine new molecular markers and potential targets for treatment [29]. Microarray data of PaCa 5061 revealed recurrent regions of amplification, including genes that are implicated in cell proliferation (RAB18, ERBB3, MAP3K12, MIB1), signal transduction (STAT2, STAT6, IGFBP6), cell cycle or cell migration (MMP19), all important genes for malignant progression. In addition to these target genes we identified an amplification of the chromosomal region 18q11.2 containing the gene for the transcription factor GATA-6, whose gain and overexpression may play an important and hitherto uninvestigated role in pancreatic carcinogenesis [30]. For some other amplified genes, MEMO1, IGFBP6, TBK1, MIB1 and RIOK3 we detected an elevated RNA expression, suggesting an important role in carcinogenesis of these cells. For some of these genes putative roles in pancreatic adenocarcinoma progression have been identified, as RIOK3 [31] and MEMO1 [32] or in tumor angiogenesis such as TBK1 [33]. Thus, the overexpression of the MEMO1 and the TBK1 genes in PaCa 5061 cells could be associated with the high tumorigenic and metastatic nature of these cells.

The elevation of gene expression promotes the optimal proliferation and survival of cancer cells in their primary environment by inhibition of apoptosis and enhancement of the cell-division cycle, angiogenesis and invasion. Among the genes specifically overexpressed in PaCa 5061 cells were numerous AKT- and MAPK- kinases, and some insulin growth factor binding proteins (IGFB-proteins), molecules with pivotal roles in signal transduction. AKT-kinases have been demonstrated to be major mediators of survival signals in a variety of cancer cells [34]. Furthermore, it was shown that AKT-kinases are capable of suppression of apoptosis in a transcription-independent manner through direct phosphorylation and inactivation of the apoptotic machinery [35,36]. In fact, the AKT gene is frequently amplified and the AKT protein is constitutively active in more than 60% of pancreatic carcinomas [37,38]. The notion that the activation of signal pathways associated with cell proliferation and resistance to apoptosis may, at least partially, explain the aggressiveness of pancreatic cancer which is supported by our findings that several mediators of such effects (amphiregulin, heparin-binding EGF like growth factor, epiregulin and vascular endothelial growth factor C) and their corresponding receptors were upregulated.

Specific features of PDAC are tumor-growth into surrounding vascular or visceral structures and an early tumor spread to distant sites. These processes require degradation of the surrounding extracellular matrix by matrix metalloproteinases (MMPs) [39,40]. Indeed, we found several MMPs (MMP-10, MMP-28) to be upregulated in PaCa 5061. Recently, MMP-10 was shown to be specifically upregulated and involved in metastatic spread to the liver in xenografts of pancreatic cancer [41]. Hence, MMPs become more and more attractive as targets for anti-cancer drugs.

The top hits of overexpressed genes (fold change > 10) were Mucin 4 (MUC4) and some family members of the carcinoembryonic antigen-related adhesion molecules (CEACAM 1, CEACAM 5 and CEACAM 6). MUC4, a high-molecular weight glycoprotein with a multidomain organization, plays multifunctional role in cell physiology [42,43]. MUC4 has already been linked to PDAC as an aberrantly expressed gene with no detectable expression in normal pancreas or chronic pancreatitis [44,45]. Recent studies have shown that MUC4 is implicated in modulation of ErbB2 signalling, in repression of apoptosis, in regulation of cell adhesion, in promoting tumor progression and metastasis, and in multidrug resistance processes [46]. The fact that elevated MUC4 expression occurs in the majority (70-80%) of pancreatic carcinomas, most notably at early onset of disease progression, leads to the discussion of MUC4 as a new tumor marker for this deadly disease [47]. Our results would corroborate this hypothesis.

To our knowledge, the simultaneous overexpression of CEACAM-1, -5 and -6 proteins in pancreatic cancer cells has not been reported before. CEACAMs belong to the family of mammalian immunoglobulin-related glycoproteins, which are involved in cell-cell recognition and modulate cellular processes that range from the shaping of tissue architecture to (tumor) neovascularization. It was shown that CEACAM expression is elevated in many solid tumors [48,49]. Functionally, CEACAMs have been implicated in cell adhesion, cellular invasiveness, resistance to anoikis and drug treatment, and to metastatic behaviour of tumor cells [50-53]. Therefore, the elevated CEACAM 1, -5 and -6 and MUC4 levels in PaCa 5061 cells may well contribute to the high metastatic potential of these cells as well.

In comparison with other gastrointestinal malignancies, pancreatic cancer seems to be one of the most resistant cancers to chemotherapy [54]. Therefore, we evaluated the cytotoxic effects of two standard chemotherapeutic drugs, Gemcitabine and 5-FU, and two targeted therapeutics, Gefinitib and Cetuximab, on PaCa 5061 cells. These drugs show a limited efficacy with regard to tumor response and prolongation of patient survival [55]. Consistently, PaCa 5061 cells were highly resistant to both, Gemcitabine and 5-FU. The molecular and biological mechanisms why most pancreatic cancer cells are insensitive to chemotherapy and escape the cytotoxic effects are largely unknown.

## Conclusion

Our newly established pancreatic cancer cell line, PaCa 5061, highly resembles the *in vivo *tumor biology of PDAC. Because of its similarity to the original human tumor, it offers an ideal *in vivo *model for the evaluation of drug-resistance and novel therapeutic approaches, in particular those which target cancer cell dissemination or metastatic spread of this deadly neoplasia.

## Competing interests

The authors declare that they have no competing interests.

## Authors' contributions

TK conceived the aims of study; performed immunohistochemistry, drug sensitivity experiments, and drafted the manuscript. CG participated in the design of the study, data analysis and manuscript preparation. MM-K and VK performed molecular genetic studies. ST, RS, TS and BO carried out the DNA and RNA profile using Affymetrix microarray technology and data annotation. EMM and JD obtained cytogenetic characteristics by karyotype analysis. DW and US carried out of the animals experiments and performed histopathology. US, HS and KE took part in data interpretation and critically revised the manuscript. MB, JRI and EFY initiated the study and participated in its design and coordination. All the authors have read and have approved of the final manuscript.

## Pre-publication history

The pre-publication history for this paper can be accessed here:

http://www.biomedcentral.com/1471-2407/10/295/prepub

## Supplementary Material

Additional file 1 **Selection of genes differentially expressed in PaCa 5061 cells vs. normal pancreas**. The gene expression profile of the PaCa 5061 cell line was compared to the RNA expression pattern obtained from normal pancreas. The fold changes of some selected genes are shown. Click here for file
