# Supplementary material for: Establishment and characterization of a new human pancreatic adenocarcinoma cell line with high metastatic potential to the lung
Source: BMC Cancer. 2010 Jun 16;10:295. doi: 10.1186/1471-2407-10-295 (PMC2927995; doi:10.1186/1471-2407-10-295)
Supplement: Additional file 1 — Selection of genes differentially expressed in PaCa 5061 cells vs. normal pancreas. The gene expression profile of the PaCa 5061 cell line was compared to the RNA expression pattern obtained from normal pancreas. The fold changes of some selected genes are shown. [file 1471-2407-10-295-S1.DOC]

**Selection of genes differentially expressed in PaCa 5061 cells *vs*. normal pancreas**

|  | **Gene symbol** | **Gene title** | **Chromosome** | **Fold change** | **Change P-value** | **Probe Set ID** |
| --- | --- | --- | --- | --- | --- | --- |
| **Signal transduction** | EGFR | epidermal growth factor receptor (erythroblastic leukemia viral (v-erb-b) oncogene homolog, avian) | 7p12 | 48.5 | 0.000023 | 211607_x_at |
|  | IGF1R | insulin-like growth factor 1 receptor | 15q26.3 | 2.8 | 0.000438 | 243358_at |
|  | IGF2BP3 | insulin-like growth factor 2 mRNA binding protein 3 | 7p11 | 181.0 | 0.00002 | 203819_s_at |
|  | IGFBP3 | insulin-like growth factor binding protein 3 | 7p13-p12 | 9.2 | 0.00002 | 212143_s_at |
|  | IGFBP6 | insulin-like growth factor binding protein 6 | 12q13 | 5.3 | 0.000027 | 203851_at |
|  | IGF2BP2 | insulin-like growth factor 2 mRNA binding protein 2 | 3q27.2 | 9.2 | 0.00002 | 218847_at |
|  | IGFBP5 | insulin-like growth factor binding protein 5 | 2q33-q36 | 4.9 | 0.000241 | 211958_at |
|  |  |  |  |  |  |  |
|  | AKTIP | AKT interacting protein | 16q12.2 | 1.5 | 0.000273 | 218373_at |
|  | AKT3 | v-akt murine thymoma viral oncogene homolog 3 (protein kinase B, gamma) | 1q43-q44 | 1.7 | 0.000088 | 222880_at |
|  | MAP3K1 | mitogen-activated protein kinase kinase kinase 1 | 5q11.2 | 14.9 | 0.00002 | 211083_s_at |
|  | MAP3K2 | mitogen-activated protein kinase kinase kinase 2 | 2q14.3 | 4.9 | 0.000035 | 221695_s_at |
|  | MAP3K7 | mitogen-activated protein kinase kinase kinase 7 | 6q16.1-q16.3 | 3.5 | 0.00002 | 211537_x_at |
|  | MAP3K8 | mitogen-activated protein kinase kinase kinase 8 | 10p11.23 | 2.0 | 0.001336 | 205027_s_at |
|  | MAP3K9 | mitogen-activated protein kinase kinase kinase 9 | 14q24.3-q31 | 3.0 | 0.000046 | 213927_at |
|  | MAP3K13 | mitogen-activated protein kinase kinase kinase 13 | 3q27 | 14.9 | 0.00002 | 211083_s_at |
|  | MAP4K5 | mitogen-activated protein kinase kinase kinase kinase 5 | 14q11.2-q21 | 21.1 | 0.000027 | 211081_s_at |
|  | MAP4K4 | mitogen-activated protein kinase kinase kinase kinase 4 | 2q11.2-q12 | 2.6 | 0.00004 | 222547_at |
|  | MAP4K3 | mitogen-activated protein kinase kinase kinase kinase 3 | 2p22.1 | 1.7 | 0.00002 | 218311_at |
|  | MAP3K7IP3 | mitogen-activated protein kinase kinase kinase 7 interacting protein 3 | Xp21.2 | 4.9 | 0.00002 | 1552928_s_at |
|  | MAP3K7IP2 | mitogen-activated protein kinase kinase kinase 7 interacting protein 2 | 6q25.1-q25.3 | 3.5 | 0.000167 | 210284_s_at |
|  | MAPK1 | mitogen-activated protein kinase 1 | 22q11.2 | 3.2 | 0.00013 | 1552264_a_at |
|  | MAPK6 | mitogen-activated protein kinase 6 | 15q21 | 2.6 | 0.00002 | 207121_s_at |
|  | MAP7D1 | MAP7 domain containing 1 | 1p34.3 | 2.1 | 0.000023 | 217943_s_at |
|  | FCF1 /// MAPK1IP1L | FCF1 small subunit (SSU) processome component homolog (S. cerevisiae) /// mitogen-activated protein kinase 1 interacting protein 1-like | 14q22.3 14q24.3 | 2.6 | 0.00002 | 212499_s_at |
|  | MBIP | MAP3K12 binding inhibitory protein 1 | 14q13.3 | 3.2 | 0.000189 | 218411_s_at |
|  |  |  |  |  |  |  |
|  | EREG | epiregulin | 4q13.3 | 55.7 | 0.000114 | 205767_at |
|  |  |  |  |  |  |  |
|  | TGFA | transforming growth factor, alpha | 2p13 | 3.7 | 0.00003 | 205016_at |
| **Angiogenesis** | IL18 | interleukin 18 (interferon-gamma-inducing factor) | 11q22.2-q22.3 | 6.5 | 0.00002 | 206295_at |
|  | IL1B | interleukin 1, beta | 2q14 | 2.8 | 0.00001 | 39402_at |
|  | IL8 | interleukin 8 | 4q13-q21 | 1.6 | 0.00013 | 202859_x_at |
|  | IL1A | interleukin 1, alpha | 2q14 | 128.0 | 0.000068 | 210118_s_at |
|  | IL15 | interleukin 15 | 4q31 | 2.8 | 0.00002 | 217371_s_at |
|  |  |  |  |  |  |  |
|  | MMP28 | matrix metallopeptidase 28 | 17q11-q21.1 | 19.7 | 0.000023 | 219909_at |
|  | MMP10 | matrix metallopeptidase 10 | 11q22.3 | 1.5 | 0.038415 | 205680_at |
|  |  |  |  |  |  |  |
| **Cell adhesion** | CEACAM1 | carcinoembryonic antigen-related cell adhesion molecule 1 | 19q13.2 | 13.0 | 0.00002 | 209498_at |
|  | CEACAM5 | carcinoembryonic antigen-related cell adhesion molecule 5 | 19q13.1-q13.2 | 194.0 | 0.000027 | 201884_at |
|  | CEACAM6 | carcinoembryonic antigen-related cell adhesion molecule 6 | 19q13.2 | 22.6 | 0.00002 | 203757_s_at |
|  |  |  |  |  |  |  |
|  | ITGBL1 | Integrin, beta-like 1 | 13q33 | 19.6 | 0.00004 | 1557079_at |
|  | ITGA2 | integrin, alpha 2 | 5q23-q31 | 22.6 | 0.00002 | 227314_at |
|  | ITGA3 | integrin, alpha 3 | 17q21.33 | 6.1 | 0.00002 | 201474_s_at |
|  | ITGB4 | integrin, beta 4 | 17q25 | 11.3 | 0.000023 | 204990_s_at |
|  | ITGA6 | integrin, alpha 6 | 2q31.1 | 3.0 | 0.000114 | 201656_at |
|  | ITGB1 | integrin, beta 1 | 10p11.2 | 4.3 | 0.00002 | 1553530_a_at |
|  | ITGB6 | integrin, beta 6 | 2q24.2 | 9.8 | 0.000023 | 208084_at |
|  | ITGB8 | integrin, beta 8 | 7p15.3 | 1.7 | 0.00002 | 226189_at |
|  | ITGAV | integrin, alpha V | 2q31-q32 | 1.5 | 0.00002 | 202351_at |
|  |  |  |  |  |  |  |
|  | CDH2 | cadherin 2, type 1, N-cadherin (neuronal) | 18q11.2 | 3.7 | 0.000273 | 203440_at |
|  | CDH3 | cadherin 3, type 1, P-cadherin (placental) | 16q22.1 | 48.5 | 0.00002 | 203256_at |
|  | PCDH1 | protocadherin 1 | 5q32-q33 | 2.3 | 0.000389 | 203918_at |
|  | PCDH7 | protocadherin 7 | 4p15 | 11.3 | 0.000273 | 205534_at |
|  | PCDHB14 | protocadherin beta 14 | 5q31 | 3.5 | 0.000046 | 231726_at |
|  | CTNNA1 | catenin (cadherin-associated protein), alpha 1, 102kDa | 5q31 | 4.3 | 0.00002 | 1558214_s_at |
|  | CTNNAL1 | catenin (cadherin-associated protein), alpha-like 1 | 9q31.2 | 3.3 | 0.00002 | 202468_s_at |
|  | CTNND1 | catenin (cadherin-associated protein), delta 1 | 11q11 | 2.8 | 0.00002 | 208407_s_at |
|  |  |  |  |  |  |  |
| **Surface** | MUC4 | mucin 4, cell surface associated | 3q29 | 776.0 | 0.00004 | 217109_at |
|  | MUC16 | mucin 16, cell surface associated | 19p13.2 | 97.0 | 0.00002 | 220196_at |
|  | MUC20 | mucin 20, cell surface associated | 3q29 | 11.3 | 0.000052 | 231941_s_at |
|  |  |  |  |  |  |  |
|  | CD9 | CD9 molecule | 12p13.3 | 2.6 | 0.00002 | 201005_at |
|  |  |  |  |  |  |  |
| **Growth factor** | AREG | amphiregulin | 4q13-q21 | 4.3 | 0.00003 | 205239_at |
|  |  |  |  |  |  |  |
|  |  |  |  |  |  |  |
|  | EREG | epiregulin | 4q13.3 | 55.7 | 0.000114 | 205767_at |
|  |  |  |  |  |  |  |
|  | VEGFC | vascular endothelial growth factor C | 4q34.1-q34.3 | -4.0 | 0.00002 | 209946_at |
|  |  |  |  |  |  |  |
| **Others** | MEMO1 | Mediator of cell motility 1 | 2p22-p21 | 3.0 | 0.000189 | 1555522_s_at |
|  |  |  |  |  |  |  |
|  | TBK1 | TANK-binding kinase 1 | 12q14.1 | 3.5 | 0.00002 | 218520_at |
|  |  |  |  |  |  |  |
|  | MIB1 | mindbomb homolog 1 (Drosophila) | 18q11.2 | 8 | 0.00002 | 224725_at |
|  |  |  |  |  |  |  |
|  | RIOK3 | RIO kinase 3 (yeast) | 18q11.2 | 4.9 | 0.00002 | 202129_s_at |
|  |  |  |  |  |  |  |
|  | EZR | ezrin | 6q25.2-q26 | 12.1 | 0.0002 | 208621_s_at |
